# Characterization of a Multidrug-Resistant Porcine Klebsiella pneumoniae Sequence Type 11 Strain Coharboring *bla*_KPC-2_ and *fosA3* on Two Novel Hybrid Plasmids

**DOI:** 10.1128/mSphere.00590-19

**Published:** 2019-09-11

**Authors:** Wanjiang Zhang, Yao Zhu, Changzhen Wang, Wenyu Liu, Ruichao Li, Fuguang Chen, Tian Luan, Yanhe Zhang, Stefan Schwarz, Siguo Liu

**Affiliations:** a State Key Laboratory of Veterinary Biotechnology, Harbin Veterinary Research Institute, Chinese Academy of Agricultural Sciences, Harbin, China; b Jiangsu Co-Innovation Center for Prevention and Control of Important Animal Infectious Diseases and Zoonoses, College of Veterinary Medicine, Yangzhou University, Yangzhou, Jiangsu, China; c Institute of Microbiology and Epizootics, Centre for Infection Medicine, Department of Veterinary Medicine, Freie Universität Berlin, Berlin, Germany; Antimicrobial Development Specialists, LLC

**Keywords:** KPC-2, carbapenem resistance, plasmid, food-producing animal

## Abstract

The occurrence of carbapenemase-producing *Enterobacteriaceae* (CPE) poses a considerable risk for public health. The gene for Klebsiella pneumoniae carbapenemase-2 (KPC-2) has been reported in many countries worldwide, and KPC-2-producing strains are mainly of human origin. In this study, we identified two novel hybrid plasmids that carry either *bla*_KPC-2_ or the fosfomycin resistance gene *fosA3* in the multiresistant K. pneumoniae isolate K15 of swine origin in China. The *bla*_KPC-2_-bearing plasmid pK15-KPC was a fusion derivative of an IncF33:A−:B− incompatibility group (Inc) plasmid and chromosomal sequences of K. pneumoniae (CSKP). A 5-bp direct target sequence duplication (GACTA) was identified at the boundaries of the CSKP, suggesting that the integration might have been due to a transposition event. The *bla*_KPC-2_ gene on pK15-KPC was in a derivative of ΔTn*6296-1*. The multireplicon *fosA3*-carrying IncN-IncR plasmid pK15-FOS also showed a mosaic structure, possibly originating from a recombination between an epidemic *fosA3*-carrying pHN7A8-like plasmid and a pKPC-LK30-like IncR plasmid. Stability tests demonstrated that both novel hybrid plasmids were stably maintained in the original host without antibiotic selection but were lost from the transformants after approximately 200 generations. This is apparently the first description of a porcine sequence type 11 (ST11) K. pneumoniae isolate coproducing KPC-2 and FosA3 via pK15-KPC and pK15-FOS, respectively. The multidrug resistance (MDR) phenotype of this high-risk K. pneumoniae isolate may contribute to its spread and its persistence.

**IMPORTANCE** The global dissemination of carbapenem resistance genes is of great concern. Animals are usually considered a reservoir of resistance genes and an important source of human infection. Although carbapenemase-producing *Enterobacteriaceae* strains of animal origin have been reported increasingly, *bla*_KPC-2_-positive strains from food-producing animals are still rare. In this study, we first describe the isolation and characterization of a carbapenem-resistant Klebsiella pneumoniae ST11 isolate, strain K15, which is of pig origin and coproduces KPC-2 and FosA3 via two novel hybrid plasmids. Furthermore, our findings highlight that this ST11 Klebsiella pneumoniae strain K15 is most likely of human origin and could be easily transmitted back to humans via direct contact or food intake. In light of our findings, significant attention must be paid to monitoring the prevalence and further evolution of *bla*_KPC-2_-carrying plasmids among the *Enterobacteriaceae* strains of animal origin.

## Abstract (precis)

The global dissemination of carbapenem resistance genes is of great concern. Animals are usually considered a reservoir of resistance genes and an important source of human infection. Although carbapenemase-producing *Enterobacteriaceae* strains of animal origin have been reported increasingly, *bla*_KPC-2_-positive strains from food-producing animals are still rare. In this study, we first describe the isolation and characterization of a carbapenem-resistant Klebsiella pneumoniae ST11 isolate, strain K15, which is of pig origin and coproduces KPC-2 and FosA3 via two novel hybrid plasmids. Furthermore, our findings highlight that this ST11 Klebsiella pneumoniae strain K15 is most likely of human origin and could be easily transmitted back to humans via direct contact or food intake. In light of our findings, significant attention must be paid to monitoring the prevalence and further evolution of *bla*_KPC-2_-carrying plasmids among the *Enterobacteriaceae* strains of animal origin.

## INTRODUCTION

Since the Klebsiella pneumoniae carbapenemase (KPC) was first identified in North Carolina in 1996 (1), KPC-producing K. pneumoniae (KPC-Kp) strains have spread globally. These strains are challenging pathogens that pose a great threat to public health, due to their multidrug resistance (MDR) phenotypes and due to significantly higher rates of morbidity and mortality associated with infections by these strains compared to the rates of morbidity and mortality associated with nonresistant bacteria (2, 3). As a member of the carbapenem-resistant *Enterobacteriaceae* (CRE), KPC-Kp was recognized as an urgent threat to public health in reports issued by the U.S. CDC and the UK Department of Health (4). Recently, the occurrence and spread of extended-spectrum β-lactamase (ESBL)-producing hypervirulent K. pneumoniae (HvKP) and KPC-2-producing HvKP have deepened our understanding of the importance of KPC-Kp (5–8). The ongoing rapid global dissemination of KPC-Kp mainly involves the dominant clonal group 258 (CG258), including the most prevalent multilocus sequence types ST258 and ST11, which prevail in different parts of the world. The horizontal transfer of KPC-encoding plasmids between bacteria of the same or different genera has also been documented (9, 10).

In contrast to the situation in humans, K. pneumoniae is widely considered an opportunistic pathogen that can inhabit the gastrointestinal tract of healthy animals, although it can also cause invasive diseases in different animal species (e.g., pig, chicken, and horse) and is a common cause of mastitis in dairy cows (11). The antimicrobial resistance of K. pneumoniae isolates of animal origin has not received much attention compared with that of other *Enterobacteriaceae*, such as Escherichia coli. However, there have been growing concerns in the veterinary field regarding the occurrence of ESBL-producing K. pneumoniae isolates in companion animals, as well as food-producing animals, in recent years (12–14). Nonetheless, KPC-Kp isolates from food-producing animals have rarely been detected so far. A few reports describe the occurrence of such isolates among broilers in Egypt (15) or functional *bla*_KPC-2_ sequences in beef cattle feces in the United States (16).

In the present study, we report for the first time the occurrence of a KPC-2- and FosA3-producing K. pneumoniae isolate, strain K15, obtained from a diseased pig in China. We further analyze in depth the structure and organization of the two plasmids that harbored the *bla*_KPC-2_ and *fosA3* genes.

## RESULTS AND DISCUSSION

### Phenotypic and genotypic characteristics of the KPC-2-producing strain.

K. pneumoniae K15 exhibited an MDR profile for a wide range of antimicrobial agents, including meropenem, cefepime, and ciprofloxacin, which are classified as critically important antimicrobials for human medicine by the World Health Organization (WHO). However, this isolate was susceptible to colistin and tetracycline (Table 1) (17). Comprehensive resistome analysis of K. pneumoniae K15 revealed the presence of β-lactam resistance genes (*bla*_KPC-2_, *bla*_CTX-M-55/-14_, and *bla*_TEM-1_) and other important resistance determinants conferring resistance to quinolones (*qnrS1* and *oqxAB*), aminoglycosides [*aadA2*, *rmtB*, and *aac(*3*)-IId*], fosfomycin (*fosA3*), chloramphenicol (*catA2*), chloramphenicol/florfenicol (*floR*), sulfonamides (*sul1*), and trimethoprim (*dfrA1*). S1 nuclease pulsed-field gel electrophoresis (S1-PFGE) and hybridization revealed two plasmids in the K15 strain, with the *bla*_KPC-2_ and *fosA3* genes being located on different plasmids of ∼180 kb (designated pK15-KPC) and ∼115 kb (designated pK15-FOS), respectively. Although conjugation experiments were unsuccessful for both plasmids, they could be transferred into E. coli strain DH5α via electrotransformation. The two transformants, TK15-KPC and TK15-FOS, exhibited substantially increased MICs for β-lactams (including meropenem) and fosfomycin, respectively (Table 1). Notably, the K15 strain belongs to the high-risk clone K. pneumoniae ST11, which is the most frequent sequence type contributing to the worldwide spread of KPC-Kp in Asia (10). This clone has also been found in Latin America and Spain (18, 19). The dominant clone K. pneumoniae ST11 mediating the spread of KPC or ESBL genes has also been detected in broilers in Egypt and China (15, 20), respectively. These findings, combined with our results, indicate that K. pneumoniae ST11 is spreading across continents and across host species.

**TABLE 1:** MICs for clinical strain K15 and its transformants TK15-KPC and TK15-FOS

Isolate	Species	Plasmid(s) harbored	MIC (mg/liter) of^*a*^:											
			MEM	FOS	CAZ	FEP	GEN	CST	CIP	FFC	CHL	TET	AMK	SXT
K15	K. pneumoniae	pK15-KPC, pK15-FOS	256	>512	256	256	>512	2	256	32	>512	2	512	>32/608
TK15-KPC	E. coli	pK15-KPC	2	16	16	2	0.5	0.25	≤0.031	4	128	0.5	2	0.063/1.19
TK15-FOS	E. coli	pK15-FOS	0.031	512	1	0.5	256	0.25	≤0.031	4	2	0.5	256	0.063/1.19
DH5α	E. coli		0.031	16	0.5	0.031	1	0.25	≤0.031	4	2	1	2	0.063/1.19

a MEM, meropenem; FOS, fosfomycin; CAZ, ceftazidime; FEP, cefepime; GEN, gentamicin; CST, colistin; CIP, ciprofloxacin; FFC, florfenicol; CHL, chloramphenicol; TET, tetracycline; AMK, amikacin; SXT, trimethoprim-sulfamethoxazole.

### Structure of the KPC-2-encoding plasmid pK15-KPC.

**FIG 1:**
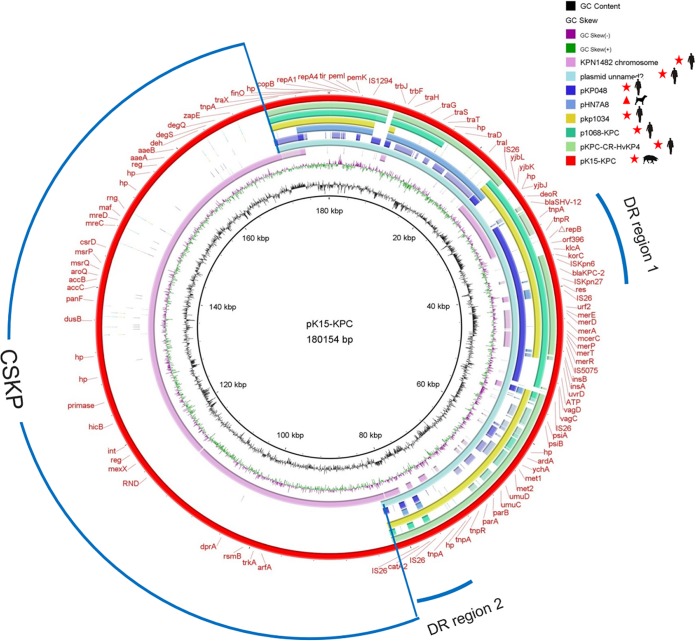
Sequence alignment of K. pneumoniae strain KPN1482 chromosome DNA (GenBank accession number NZ_CP020841), plasmid unnamed 2 (GenBank accession number CP023938), pKP048 (GenBank accession number FJ628167), pHN7A8 (GenBank accession number JN232517), pKP1034 (GenBank accession number KP893385), p1068-KPC (GenBank accession number MF168402), pKPC-CR-HvKP4 (GenBank accession number MF437312), and pK15-KPC (GenBank accession number MK433207). pK15-KPC was used as a reference to compare with the strain KPN1482 chromosome, plasmid unnamed 2, pKP048, pHN7A8, pKP1034, p1068-KPC, and pKPC-CR-HvKP4. The red outer circle denotes annotation of the reference plasmid. The circles show (from outside to inside): predicted coding sequences (CDS), GC skew, GC content, and scale in kilobase pairs. CSKP represents the chromosomal sequences of K. pneumoniae. Red star, plasmid was isolated from a K. pneumoniae strain; red triangle, plasmid was isolated from an E. coli strain.

Plasmid pK15-KPC is 180,154 bp in size, has an average GC content of 54.6%, and contains 125 open reading frames (ORFs), only 33 of which encode proteins with known functions, such as plasmid replication, transfer, or maintenance or antimicrobial resistance (Table S1 in the supplemental material). pK15-KPC belongs to the IncF33:A−:B− incompatibility (Inc) group. The overall genetic structure of pK15-KPC is a fusion derived from a plasmid and chromosomal sequences. It can be divided into two genetically distinct modules: (i) a 90,244-bp plasmid backbone and (ii) an 89,905-bp fragment that contains chromosomal sequences of K. pneumoniae (CSKP) (Fig. 1).

10.1128/mSphere.00590-19.1 TABLE S1 Annotation of ORFs in plasmid pK15-KPC. Download Table S1, DOCX file, 0.04 MB. Copyright © 2019 Zhang et al. 2019 Zhang et al. This content is distributed under the terms of the Creative Commons Attribution 4.0 International license.

Except for the CSKP fragment, pK15-KPC shows high homology to an unnamed F33:A−:B− *bla*_KPC-2_-carrying plasmid (GenBank accession number CP023942) from human K. pneumoniae strain FDAARGOS_444, isolated in the United States. We observed 100% query coverage and 99% nucleotide identity. Additionally, 85% and 74% query coverage and 99% nucleotide similarities were observed when comparing pK15-KPC with the *bla*_KPC-2_-harboring plasmid pKPC-CR-HvKP4 from a carbapenem-resistant hypervirulent ST11 K. pneumoniae strain in China (7) and plasmid pKP1034 (GenBank accession number KP893385) coharboring *bla*_KPC-2_, *fosA3*, *rmtB*, and *bla*_CTX-M-65_ from an ST11 K. pneumoniae strain in China, both of which were multireplicon plasmids carrying IncF33:A−:B− and IncR replicons.

Comparative analysis of the replication region (composed of *repA1*, *repA2*, and *repA4* genes) and the transfer region (comprising *trbJ*, *trbF*, and *traHGSTDI* genes) of pK15-KPC showed that the two regions were organized very similarly to K. pneumoniae plasmids pKP1034, p1068-KPC, and pKPC-CR-HvKP4, as well as the E. coli plasmid pHN7A8, an F33:A−:B− type epidemic plasmid cocarrying *fosA3*, *bla*_CTX-M-65_, *rmtB*, and *bla*_TEM-1_ genes (21). However, the *tra* region in pK15-KPC is incomplete compared with that in pHN7A8, and the deleted part of the *tra* region in pK15-KPC is occupied by Δ*tnpA* of Tn*2*, an IS*1294* element, a putative ORF encoding a phage integrase, and an IS*26* element. The deletion of the *tra* region in pK15-KPC may explain why pK15-KPC was not able to transfer conjugatively to E. coli strain J53, as was pHN7A8. Another two pHN7A8-related multiresistance plasmids coharboring the *bla*_CTX-M-65_, *fosA3*, and *rmtB* genes, p397Kp and p477Kp, were detected in human clinical isolates of K. pneumoniae from Bolivia in 2016 (22), indicating intercontinental dissemination of pHN7A8-like plasmids. Altogether, these results, combined with our findings in this study, suggest that genetic recombination or extensive gene exchange events can readily occur between pHN7A8 and other plasmids of different incompatibility groups, including pK15-KPC.

### Multidrug resistance region of the KPC-2-encoding plasmid pK15-KPC.

**FIG 2:**
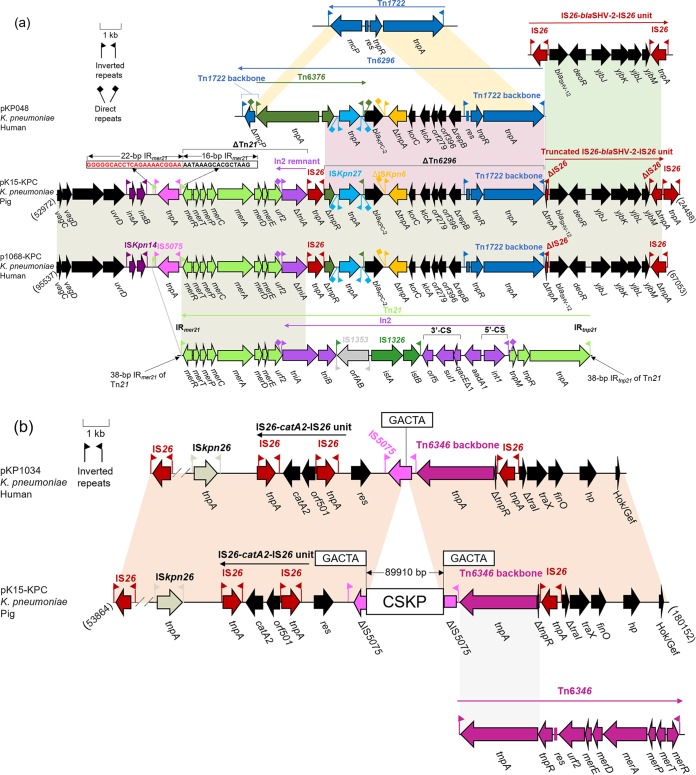
(a) Organization of the plasmid pK15-KPC MDR region. The MDR region of plasmid pK15-KPC is compared with Tn*1722* (GenBank accession number X61367), pKP048 (GenBank accession number FJ628167), p1068-KPC (GenBank accession number MF168402), and Tn*21* (GenBank accession number AF071413). The 38-bp IR*_mer21_* of the Tn*21* sequence is boxed. (b) Linear comparison of the region of plasmid pK15-KPC in which CSKP is inserted and plasmid pKP1034 (GenBank accession number KP893385). The 5-bp direct target site duplication sequences (GACTA) of CSKP are boxed. Genes are denoted by arrows and are colored based on gene function classification. Shaded regions denote shared regions of homology (>95% nucleotide identity). The scale of identity is shown on the left.

The multidrug resistance (MDR) plasmid pK15-KPC harbors three antibiotic resistance genes located in two drug resistance (DR) regions (Fig. 1). The primary components of the 22.4-kb DR region 1 consist of ΔTn*21*, containing an intact mercury resistance operon and a 1,210-bp remnant of the In*2* class 1 integron *tniA* gene, ΔTn*6296*, carrying the *bla*_KPC-2_ gene, and one IS*26*-based transposition unit composed of ΔIS*26*-*bla*_SHV-12_-*deoR*-*yjbJ*-*yjbK*-*yjbM*-ΔIS*26* (Fig. 2a). As reported in Europe and the Americas, the most common *bla*_KPC-2_-containing mobile element is a Tn*3* family transposon named Tn*4401.* Its core structure is Tn*3*-IS*Kpn7*-*bla*_KPC-2_-IS*Kpn6*, which is mainly carried by Inc group FII plasmids, as well as a variety of other plasmids of different Inc groups, such as FIA, I2, A/C, N, X, R, P, U, W, L/M, and ColE (3). However, the *bla*_KPC-2_ gene found in isolates from China is exclusively located in the novel transposon Tn*6296* and its derivatives (23). Archetypal Tn*6296*, as observed in the MDR plasmid pKP048, derived from the ST11 K. pneumoniae isolate KP048 in China (24), is formed by the insertion of a core *bla*_KPC-2_ module (Tn*6376*-*bla*_KPC-2_-ΔIS*Kpn6*-*korC*-*klcA*-*orf279*-*orf396*-Δ*repB*) that has been integrated into Tn*1722*, thereby resulting in the truncation of the gene *mcP* (Fig. 2a). To date, at least four Tn*6296* derivatives resulting from insertions, deletions, and rearrangements at different locations have been reported in KPC-producing plasmids from human K. pneumoniae isolates in China (23). Based on the above-mentioned classification criteria (23), the *bla*_KPC-2_ gene in pK15-KPC is in the ΔTn*6296-1* derivative that lacks a 3,804-bp region including the Tn*6376*-associated *tnpA* gene and Δ*mcP* (Fig. 2a). Such a structure is also found in the two K. pneumoniae plasmids pKPC-LK30 (GenBank accession number KC405622) and p1068-KPC (GenBank accession number MF168402).

Immediately downstream from the 5′ end of ΔTn*6296* of pK15-KPC, a truncated IS*26*-based transposition unit harboring the β-lactam resistance gene *bla*_SHV-2_ was detected, which is also found in p1068-KPC from a human K. pneumoniae strain in China. Nonetheless, the truncated IS*26*-based transposition unit in pK15-KPC differs from its counterpart in p1068-KPC by the absence of 272 bp of the 5′ end of the IS*26* element, which is due to the insertion of another IS*26* element. Upstream from the IS*26* element, adjacent to the 5′ end of ΔTn*6296*, are a 3′ terminal remnant of ΔTn*21* containing the 16-bp inverted repeat IR*_mer21_* of Tn*21*, a mercury resistance operon (*merRTPCADE*), and Δ*tniA*.

DR region 2 comprises the chloramphenicol resistance gene *catA2* flanked by two directly oriented IS*26* elements (Fig. 1 and 2b). This has commonly been found on different Inc group plasmids, such as p64917-KPC (IncFII and IncR) (GenBank accession number MF168405) from a human K. pneumoniae isolate and pIMP-4-EC62 (IncHI2) (GenBank accession number MH829594) from a swine Enterobacter cloacae isolate, indicating that it is horizontally transferred via plasmids that vary regarding replicon type, source, and size.

### The chromosomal insert in pK15-KPC.

**FIG 3:**
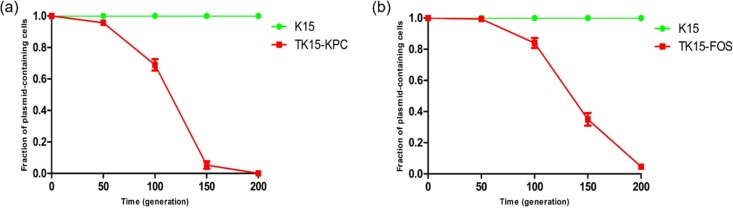
Measurement of pK15-KPC (a) and pK15-FOS (b) stability in donor and transformants. Serial passaging in antibiotic-free LB broth was performed daily. At 0, 50, 100, 150, and 200 generations, samples were tested. The *y* axis shows the percentages of cells containing the plasmid in all picked cells. Data points and error bars represent mean values ± standard deviations (SD) of three independent lineages.

The remaining region (nt 53864 to 180152) in pK15-KPC shares high identity with the corresponding fragment in the IncR-F33:A−:B− plasmid pKP1034 (Fig. 2b). However, the insertion of the 89,905-bp CSKP in the IS*5075* element in pK15-KPC was not present in pKP1034. The 5-bp direct target sequence repeat (target site duplication [TSD]) 5′-GACTA-3′ at the boundaries of the CSKP may point toward a probable integration by transposition (Fig. 2b). Notably, the same chromosomal fragment has been present not only in the chromosomal DNA of human K. pneumoniae strains from different countries, such as the WCHKP015625 strain (China, GenBank accession number CP033396) and FDAARGOS_443 strain (United States, GenBank accession number CP023933), but also on the chromosome of K15, further supporting its integrative nature. Apart from two resistance-related ORFs encoding the 16S rRNA methyltransferase RsmB and the multidrug efflux transporter permease (resistance-nodulation-division [RND] family efflux pump), the CSKP also contained genes that code for products related to the toxin-antitoxin system HicB, the AaeAB efflux system, and the DNA-protecting protein DprA, all of which likely contribute to the stability and maintenance of pK15-KPC in the parental strain K15 (Fig. 3a). Chromosomal fragments have sporadically been inserted into plasmids, thereby generating hybrid elements like the IncP-2 plasmid pJB37 from Pseudomonas aeruginosa (25). To the best of our knowledge, this is the first report of such a large chromosomal fragment being integrated into a plasmid.

### Structure and MDR region of the FosA3-encoding plasmid pK15-FOS.

**FIG 4:**
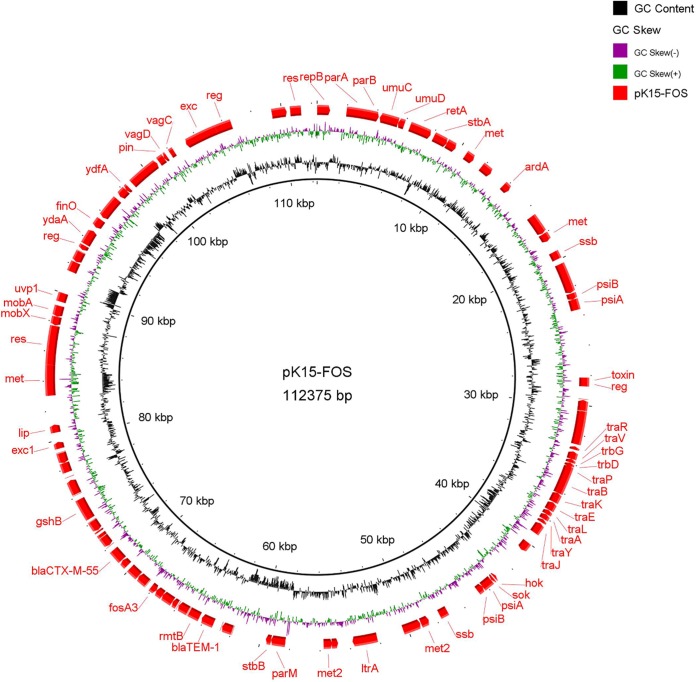
Gene map of *fosA3*-carrying plasmid pK15-FOS. GC content and GC skew are indicated from the inside out. Positions and transcriptional directions of the ORFs are indicated by arrows.

**FIG 5:**
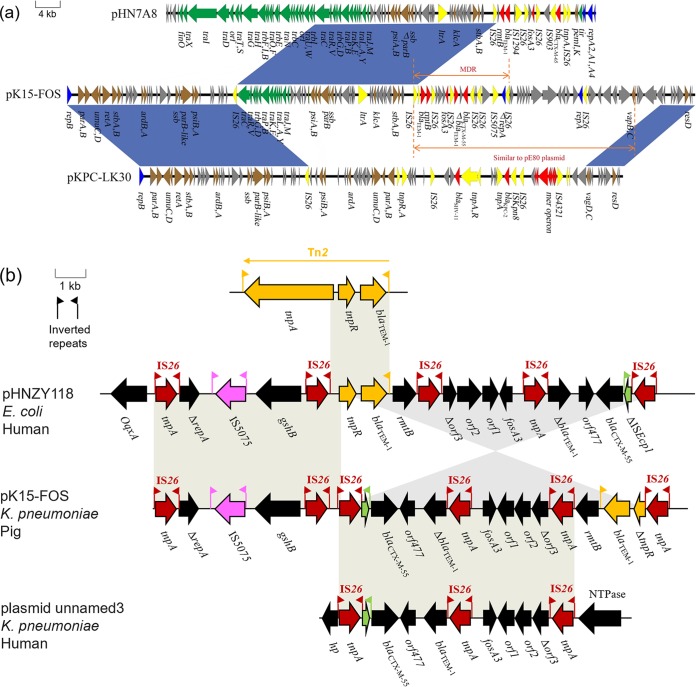
(a) Linear comparison of the complete sequences of plasmids pK15-FOS (GenBank accession number MK433206), pHN7A8 (GenBank accession number JN232517), and pKPC-LK30 (GenBank accession number KC405622). Blue shading indicates shared regions with a high degree of homology. Genes are represented by arrows and are colored depending on gene function. Genes are color coded as follows: dark blue, replication; green, conjugative transfer; brown, stability; red, antimicrobial resistance; yellow, mobile element; gray, hypothetical proteins. The scale of identity is shown on the left. (b) A comparative analysis of the complex MDR region of pK15-FOS, Tn*2* (accession number HM749967), plasmid pHNZY118 (accession number MG197503), and plasmid unnamed3 (accession number CP023934). Genes are displayed by arrows and are colored depending on gene function classification. Vertical lines represent the IRs of ISs or transposon Tn*2*.

Plasmid pK15-FOS has a size of 112,375 bp and an average GC content of 51.9% (Fig. 4). There are 87 predicted ORFs, 48.2% of which encode hypothetical proteins (Table S2). pK15-FOS has two replicons, an IncR replicon and an IncN replicon. BLASTn comparisons revealed that the backbone of pK15-FOS is highly homologous to that of IncR plasmids, such as the K. pneumoniae plasmids pKPC-LK30 (GenBank accession number KC405622) from Taiwan, pKP1034 (GenBank accession number KP893385) from China, and pKP1780 (GenBank accession number JX424614) from Greece (Fig. 5a). Its backbone regions harbor the genes *repB*, *resD*, *parAB*, *umuCD*, and *retA*, which are involved in plasmid replication, partitioning, maintenance, and stability. Sequence analysis revealed that the multidrug resistance plasmid pK15-FOS carries five antimicrobial resistance genes, including *fosA3*, *bla*_CTX-M-55_, *rmtB*, and an intact and a truncated *bla*_TEM-1_, all included in a single MDR region. In addition, six copies of intact IS*26* were found to at different sites of the MDR region. Because no TSD was found flanking IS*26*, it is possible that the generation of this MDR region was driven by IS*26*-mediated homologous recombination events. Linear comparisons demonstrated that an ∼32-kb segment immediately upstream from the MDR region exhibits high nucleotide sequence identity with the corresponding region of the epidemic IncF33:A−:B− type *fosA3*-carrying plasmid pHN7A8, isolated from an E. coli strain from a dog in China (Fig. 5a) (21). The pHN7A8-like fragment contains a partial *tra* region and several genes related to plasmid maintenance and stability, such as *psiAB*, *parB*, *ssb*, and *stbAB*. The incomplete transfer region likely explains why plasmid pK15-FOS is nonconjugative.

10.1128/mSphere.00590-19.2 TABLE S2 Annotation of ORFs in plasmid pK15-FOS. Download Table S2, DOCX file, 0.03 MB. Copyright © 2019 Zhang et al. 2019 Zhang et al. This content is distributed under the terms of the Creative Commons Attribution 4.0 International license.

Plasmid pK15-FOS exhibits a highly mosaic structure, suggesting that it has possibly undergone multiple recombination events. The analysis of the genetic environment revealed that the *fosA3* gene is in an IS*26*-based composite transposon (IS*26*-*fosA3*-*orf1*-*orf2*-Δ*orf3*-IS*26*) that has been identified in plasmids of diverse replicons, such as IncHI2, IncN, and IncF (26, 27). When compared with plasmid pHNZY118 (GenBank accession number MG197503), isolated from an E. coli strain of human origin, the MDR region shares high similarity (Fig. 5b). However, the downstream region of IS*26* adjacent to *gshB* is in the opposite orientation. Upstream from *rmtB*, a remnant of Tn*2*, including *bla*_TEM-1_ and Δ*tnpR*, was found. Moreover, the ∼7.6-kb segment downstream from *rmtB* and IS*26* adjacent to IS*Ecp1* exhibits a high degree of identity with the corresponding region of another unnamed plasmid (GenBank accession number CP023934), isolated from a K. pneumoniae strain of human origin. Whether this structural unit flanked by IS*26* is able to form more complicated IS*26*-based composite transposons requires further research.

Although fosfomycin is not used in food-producing animals in China, an increasing prevalence of *fosA3* in bacteria of animal origins has been reported (27, 28). Overall, the coexistence of *fosA3* with other resistance genes on the same plasmid may result in the persistence and dissemination of *fosA3* in food-producing animals, even in the absence of a direct selection pressure. IncR plasmids are closely associated with the spread of clinically important resistance genes, including *bla*_KPC-2_, *bla*_NDM-1_, *bla*_VIM-1_, *bla*_CTX-M_, and *armA* (29–31). Despite their inability to transfer by conjugation, IncR plasmids can broaden their host range and enhance mobility by fusion with other types of plasmids, such as IncFII, IncN, and IncA/C (31–33). Furthermore, plasmid stability experiments showed that pK15-FOS was also stably maintained in K. pneumoniae K15 (Fig. 3b), even though it was unstable in the E. coli transformant. This may have limited the spread of this plasmid between different bacterial species. However, pK15-FOS might become another important vehicle for and play a vital role in the dissemination of antimicrobial resistance genes like *fosA3* and *bla*_CTX-M-55_ in K. pneumoniae.

Until now, there have been some reports about human isolates of K. pneumoniae coharboring KPC-2 and FosA3 (34–36). To the best of our knowledge, this is the first report of an ST11 KPC-carrying K. pneumoniae isolate coproducing KPC-2 and FosA3 being recovered from a pig, specifically, from a lung sample of a diseased pig in China. From a One Health perspective, colocalization of these two genes in a single isolate of food-producing-animal origin will pose a challenge to public health. Considering the absence of carbapenem use in food-producing animals, the genotype and antibiotic resistance pattern of strain K15, and the *bla*_KPC-2_-harboring ΔTn*6296* transposon in pK15-KPC, this isolate is most likely of human origin. A serious finding is that this isolate carries 14 resistance genes, 7 of which are plasmid borne. The copresence of many resistance genes in a single strain provides this isolate with the selective advantage needed to successfully spread or persist in the animal or the farm environment. It cannot be excluded that this isolate may spread to humans via direct contact or the food chain. As such, further studies are needed to investigate the prevalence of *bla*_KPC_ genes among Gram-negative bacteria of animal origin.

## MATERIALS AND METHODS

### Bacterial isolate and antibiotic susceptibility testing.

During a surveillance study on carbapenem resistance in *Klebsiella* spp. of swine origin in China from July 2017 to June 2018, 103 *Klebsiella* species isolates were obtained from 351 swine clinical samples (278 pathological lung specimens and 73 nasal swabs). Resistance to meropenem was tested by growth on MacConkey agar plates containing 2 mg/liter meropenem for 18 h at 37°C. A single meropenem-resistant isolate, K15, was identified, and the species was confirmed using an API 20E strip (bioMérieux, Marcy-l’Étoile, France) and 16S rRNA gene sequencing (37). Multilocus sequence typing (MLST) of K. pneumoniae was then performed according to a published protocol (38). The isolate was screened for the presence of major carbapenemase genes by PCR and sequencing of the amplicons, as described previously (39). The MICs of the original isolate and its transformants were determined using the broth microdilution and agar dilution methods according to CLSI recommendations (40). The MICs of fosfomycin were determined by the agar dilution method on Mueller-Hinton (MH) agar supplemented with 25 μg/ml glucose 6-phosphate. E. coli ATCC 25922 served as a quality control strain.

### Plasmid analysis, S1-PFGE, and Southern blot hybridization.

Plasmid profiles were prepared as previously described (26, 41). Electrotransformation and conjugal transfer of the plasmids were performed using E. coli strains DH5α and J53 as recipients for the selection of *bla*_KPC-2_- or *fosA3*-positive transformants and transconjugants, respectively (26, 41). S1-PFGE and hybridization with *bla*_KPC-2_ and *fosA3* probes were employed for plasmid profiling and determining the locations of the above-mentioned resistance genes (26, 41).

### Plasmid sequencing and bioinformatics analysis.

To gain insight into the resistome of K. pneumoniae K15 and the genetic environment of *bla*_KPC-2_ and *fosA3* on the two plasmids, the draft genome sequence of K. pneumoniae K15 and the complete sequences of the two plasmids, obtained from the corresponding transformants, were determined using the Illumina NextSeq 500 and the PacBio RSII system (Tianjin Biochip Corporation, Tianjin, China). RAST combined with BLASTP/BLASTN was applied for annotating the two plasmid sequences. The resistome, MLST, and plasmid replicon typing were analyzed using bioinformatics software available from the Center for Genomic Epidemiology (http://www.genomicepidemiology.org). The BLAST Ring Image Generator (BRIG) tool was applied to compare plasmids.

### Plasmid stability tests.

The stability of two hybrid plasmids in the parental strain K. pneumoniae K15 and its E. coli transformants TK15-KPC (harboring pK15-KPC) and TK15-FOS (harboring pK15-FOS) was evaluated by passaging in antibiotic-free Luria-Bertani (LB) broth, as described previously (42).

### Accession numbers.

The complete nucleotide sequences of plasmids pK15-KPC and pK15-FOS have been deposited in GenBank under accession numbers MK433207 and MK433206, respectively.
