# Supplementary material for: Characterization of a Multidrug-Resistant Porcine Klebsiella pneumoniae Sequence Type 11 Strain Coharboring blaKPC-2 and fosA3 on Two Novel Hybrid Plasmids
Source: mSphere. 2019 Sep 11;4(5):e00590-19. doi: 10.1128/mSphere.00590-19 (PMC6739495; doi:10.1128/mSphere.00590-19)
Supplement: TABLE S1 [file mSphere.00590-19-st001.docx]

Table S1. Annotation of ORFs in plasmid pK15-KPC

| **Gene designation** | **location (start-end)** | **size (aa^a^)** | **aa identity (%)** | **Alignment region of the match^b^** | **homologue description** |
| --- | --- | --- | --- | --- | --- |
| *copB* | 109-357 | 82 | 100 | 1-82/82 | copy number control protein CopB [*K. pneumoniae*] |
| *repA1* | 657-1526 | 289 | 100 | 1-289/289 | plasmid replication initiation protein RepA1[*K. peumoniae*] |
| *repA4* | 1889-2275 | 128 | 100 | 1-128/128 | replication initiation protein RepA4 [*Klebsiella pneumoniae*] |
| *tir* | 2465-3118 | 217 | 100 | 1-217/217 | CPBP family intramembrane metalloprotease [*K. pneumoniae*] |
| *pemI* | 3211-3468 | 85 | 100 | 1-85/85 | putative plasmid stable inheritance protein I [*K. pneumoniae*] |
| *pemK* | 3470-3802 | 110 | 100 | 1-110/110 | mRNA interferase PemK [*K. pneumoniae*] |
| *tnpA* | 5938-4739 | 399 | 100 | 1-399/399 | Transposase of IS*1294* [*K. pneumoniae*] |
| *tnpA* | 8389-9093 | 238 | 100 | 1-238/238 | IS*26* transposase [*Klebsiella pneumoniae*] |
| *trbJ* | 9201-9515 | 104 | 100 | 1-104/104 | conjugal transfer protein TrbJ [*Klebsiella pneumoniae*] |
| *trbF* | 9469-9861 | 130 | 100 | 1-130/130 | conjugal transfer protein TrbF [*Klebsiella pneumoniae*] |
| *traH* | 9848-11221 | 457 | 100 | 1-457/457 | conjugal transfer protein TraH [*Klebsiella pneumoniae*] |
| *traG* | 11218-14034 | 938 | 100 | 1-938/938 | conjugal transfer protein TraG [*Klebsiella pneumoniae*] |
| *traS* | 14067-14588 | 173 | 100 | 1-173/173 | transmembrane conjugal transfer TraS [*Klebsiella pneumoniae*] |
| *traT* | 14610-15344 | 244 | 100 | 1-244/244 | transmembrane conjugal transfer TraT [*Klebsiella pneumoniae*] |
| *hp1* | 15547-16284 | 245 | 100 | 1-245/245 | hypothetical protein [*Klebsiella pneumoniae*] |
| *traD* | 16335-18551 | 738 | 100 | 1-738/738 | conjugal transfer protein TraD [*Klebsiella pneumoniae*] |
| *traI* | 18551-23686 | 1712 | 99 | 1-1712/1712 | conjugal transfer protein TraI [*Klebsiella pneumoniae*] |
| *tnpA* | 24436-23720 | 238 | 100 | 1-238/238 | IS*26* transposase [*Klebsiella pneumoniae*] |
| *yjbL* | 25977-25354 | 207 | 100 | 1-293/293 | YjbL protein [*Klebsiella pneumoniae*] |
| *yjbK* | 26608-25727 | 293 | 100 | 1-293/293 | YjbK protein [*Klebsiella pneumoniae*] |
| *yjbJ* | 27522-26620 | 300 | 100 | 1-300/300 | 3-hydroxyisobutyrate dehydrogenase [*Klebsiella pneumoniae*] |
| *deoR* | 27784-28545 | 253 | 100 | 1-253/253 | DeoR transcriptional regulator [*Klebsiella pneumoniae*] |
| *bla*_SHV-12_ | 29426-28566 | 286 | 100 | 1-286/286 | SHV-12 beta-lactamase [*Klebsiella pneumoniae*] |
| *tnpA* | 32633-29667 | 988 | 100 | 1-988/988 | Tn*1722* transposase [*Klebsiella pneumoniae*] |
| *tnpR* | 33197-32637 | 186 | 100 | 1-186/186 | Resolvase [*Klebsiella pneumoniae*] |
| △*repB* | 33646-34206 | 186 | 100 | 1-186/186 | Truncated replication protein RepB [*Klebsiella pneumoniae*] |
| *orf396* | 34361-34756 | 131 | 100 | 1-131/131 | hypothetical protein [*Klebsiella pneumoniae*] |
| *klcA* | 35129-35554 | 141 | 100 | 1-141/141 | antirestriction protein KlcA (plasmid) [*Klebsiella pneumoniae*] |
| *korC* | 35883-36179 | 98 | 100 | 1-98/98 | Transcriptional repressor KorC [*Klebsiella pneumoniae*] |
| △*tnpA* | 36184-37164 | 326 | 100 | 1-326/326 | truncated transposase IS*Kpn6* [*Klebsiella pneumoniae*] |
| *bla*_KPC-2_ | 38295-37414 | 293 | 100 | 1-293/293 | class A beta-lactamase KPC-12 [*Klebsiella pneumoniae*] |
| *tnpA* | 39551-38571 | 326 | 100 | 1-326/326 | IS*Kpn27* transposase [*Klebsiella pneumoniae*] |
| *res* | 40075-39674 | 133 | 100 | 1-133/133 | resolvase [*Klebsiella pneumoniae*] |
| *tnpA* | 40903-40187 | 238 | 100 | 1-238/238 | IS*26* transposase [*Klebsiella pneumoniae*] |
| *urf2* | 42910-42203 | 235 | 100 | 1-235/235 | Tn*21* protein of unknown function Urf2[*K. pneumoniae*] |
| *merE* | 43143-42907 | 78 | 100 | 1-78/78 | mercury resistance protein MerE [*Klebsiella pneumoniae*] |
| *merD* | 43502-43140 | 120 | 100 | 1-120/120 | transcriptional regulator MerD [*Klebsiella pneumoniae*] |
| *merA* | 45214-43520 | 564 | 100 | 1-564/564 | putative mercuric reductase [*Klebsiella pneumoniae*] |
| *merC* | 45688-45266 | 140 | 100 | 1-140/140 | mercury transport protein MerC [*Klebsiella pneumoniae*] |
| *merP* | 45999-45724 | 91 | 100 | 1-91/91 | mercuric transport protein MerP [*Klebsiella pneumoniae*] |
| *merT* | 46363-46013 | 116 | 100 | 1-116/116 | mercuric transport protein MerT [*Klebsiella pneumoniae*] |
| *merR* | 46435-46869 | 144 | 100 | 1-144/144 | transcriptional regulator MerR [*Klebsiella pneumoniae*] |
| *tnpA* | 46948-47952 | 334 | 100 | 1-334/334 | IS*5075* transposase [*Klebsiella pneumoniae*] |
| *insB* | 48910-48533 | 125 | 100 | 1-125/125 | IS*KPn14* transposase InsB [*Klebsiella pneumoniae*] |
| *insA* | 49230-48955 | 91 | 100 | 1-91/91 | IS*KPn14* transposase InsA [*Klebsiella pneumoniae*] |
| *uvrD* | 50508-49486 | 340 | 100 | 1-340/340 | Helicase UvrD [*Klebsiella pneumoniae*] |
| *ATP* | 52055-50493 | 520 | 100 | 1-520/520 | ATP-dependent endonuclease [*Klebsiella pneumoniae*] |
| *vagD* | 52545-52129 | 138 | 100 | 1-138/138 | Virulence-associated protein VagD [*Klebsiella pneumoniae*] |
| *vagC* | 52772-52542 | 76 | 100 | 1-76/76 | Virulence-associated protein VagC [*Klebsiella pneumoniae*] |
| *tnpA* | 54604-53900 | 238 | 100 | 1-238/238 | IS*26* transposase [*Klebsiella pneumoniae*] |
| *psiA* | 57199-56471 | 242 | 100 | 1-242/242 | PsiA protein [*Klebsiella pneumoniae*] |
| *psiB* | 57627-57196 | 143 | 100 | 1-143/143 | PsiB protein [*Klebsiella pneumoniae*] |
| *hp2* | 61212-60958 | 84 | 100 | 1-84/84 | DNA polymerase III theta subunit [*Klebsiella pneumoniae*] |
| *ardA* | 62145-61639 | 168 | 100 | 1-168/168 | ArdA protein [*Klebsiella pneumoniae*] |
| *ychA* | 63329-62550 | 259 | 100 | 1-259/259 | YchA [*Klebsiella pneumoniae*] |
| *met1* | 63802-63383 | 139 | 100 | 1-139/139 | Adenine-specific methyltransferase [*Klebsiella pneumoniae*] |
| *met2* | 64711-64034 | 225 | 100 | 1-225/225 | Adenine-specific methyltransferase [*Klebsiella pneumoniae*] |
| *umuD* | 65921-66343 | 140 | 100 | 1-140/140 | UmuD [*Klebsiella pneumoniae*] |
| *umuC* | 66343-67614 | 423 | 100 | 1-423/423 | UmuC [*Klebsiella pneumoniae*] |
| *parB* | 68721-67750 | 323 | 100 | 1-323/323 | Chromosome partitioning protein [*K. pneumoniae*] |
| *parA* | 69860-68718 | 380 | 100 | 1-380/380 | Chromosome partitioning protein [*K. pneumoniae*] |
| *tnpR* | 70286-70918 | 210 | 100 | 1-210/210 | putative resolvase [*K. pneumoniae*] |
| *tnpA* | 71319-72269 | 316 | 100 | 1-316/316 | putative transposase [*K. pneumoniae*] |
| *hp3* | 72877-72266 | 203 | 100 | 1-203/203 | hypothetical protein [*K. pneumoniae*] |
| *tnpA* | 73783-74763 | 326 | 100 | 1-326/326 | putative transposase [*K. pneumoniae*] |
| *tnpA* | 76429-77145 | 238 | 100 | 1-238/238 | IS*26* transposase [*K. pneumoniae*] |
| *catA2* | 77920-77279 | 213 | 100 | 1-213/213 | Chloramphenicol acetyltransferase [*K. pneumoniae*] |
| *tnpA* | 78638-79354 | 238 | 100 | 1-238/238 | IS*26* transposase [*K. pneumoniae*] |
| S10(Chro) | 83363-83674 | 103 | 100 | 1-103/103 | 30S ribosomal protein S10 [*K. pneumoniae*] |
| L3 | 83707-84336 | 209 | 100 | 1-209/209 | 50S ribosomal protein L3 [*K. pneumoniae*] |
| L4 | 84347-84952 | 201 | 100 | 1-201/201 | 50S ribosomal protein L3 [*K. pneumoniae*] |
| L23 | 84949-85251 | 100 | 100 | 1-100/100 | 50S ribosomal protein L23 [*K. pneumoniae*] |
| L2 | 85269-86090 | 273 | 100 | 1-273/273 | 50S ribosomal protein L2 [*K. pneumoniae*] |
| S19 | 86107-86385 | 92 | 100 | 1-92/92 | 30S ribosomal protein S19 [*Klebsiella pneumoniae*] |
| *rplV* | 86400-86732 | 110 | 100 | 1-110/110 | 50S ribosomal protein L22 [*Klebsiella pneumoniae*] |
| S3 | 86750-87448 | 232 | 100 | 1-232/232 | 30S ribosomal protein S3 [*Klebsiella pneumoniae*] |
| L16 | 87461-87871 | 136 | 100 | 1-136/136 | 50S ribosomal protein L16 [*Klebsiella pneumoniae*] |
| L29 | 87871-88062 | 63 | 100 | 1-63/63 | 50S ribosomal protein L29 [*Klebsiella pneumoniae*] |
| L14 | 88482-88853 | 123 | 100 | 1-123/123 | 50S ribosomal protein L14 [*Klebsiella pneumoniae*] |
| L24 | 88864-89178 | 104 | 100 | 1-104/104 | 50S ribosomal protein L24 [*Klebsiella pneumoniae*] |
| L5 | 89193-89732 | 179 | 100 | 1-179/179 | 50S ribosomal protein L5 [*Klebsiella pneumoniae*] |
| S14 | 89747-90052 | 101 | 100 | 1-101/101 | 30S ribosomal protein S14 [*Klebsiella pneumoniae*] |
| *rpsH* | 90086-90478 | 101 | 100 | 1-101/101 | 30S ribosomal protein S8 [*Klebsiella pneumoniae*] |
| L6 | 90491-91024 | 177 | 100 | 1-177/177 | 50S ribosomal protein L6 [*Klebsiella pneumoniae*] |
| L18 | 91034-91387 | 117 | 100 | 1-117/117 | 50S ribosomal protein L18 [*Klebsiella pneumoniae*] |
| *rpsE* | 91402-91905 | 167 | 100 | 1-167/167 | 30S ribosomal protein S5 [*Klebsiella pneumoniae*] |
| L15 | 92092-92526 | 144 | 100 | 1-144/144 | 50S ribosomal protein L15 [*Klebsiella pneumoniae*] |
| *rplQ* | 96634-97020 | 128 | 100 | 1-128/128 | 50S ribosomal protein L17 [*Klebsiella pneumoniae*] |
| *arfA* | 98610-98197 | 137 | 100 | 1-137/137 | ribosome rescue factor ArfA [*Klebsiella pneumoniae*] |
| *trkA* | 100127-98751 | 458 | 100 | 1-458/458 | Trk system transporter TrkA [*Klebsiella pneumoniae*] |
| *rsmB* | 101436-100141 | 431 | 100 | 1-431/431 | 16S rRNA-methyltransferase RsmB [*K. pneumoniae*] |
| *dprA* | 103089-104213 | 374 | 100 | 1-374/374 | DNA-protecting protein DprA [*K. pneumoniae*] |
| RND | 116830-113720 | 1036 | 100 | 1-1036/1036 | multidrug efflux transporter permease [*K. pneumoniae*] |
| *mexX* | 117982-116843 | 379 | 100 | 1-379/379 | MexX family efflux pump subunit [*K. pneumoniae*] |
| *reg* | 118361-119011 | 216 | 100 | 1-216/216 | acrEF/envCD transcriptional regulator [*K. pneumoniae*] |
| *int* | 119287-120513 | 408 | 100 | 1-408/408 | Integrase [*K. pneumoniae*] |
| *hicB* | 123121-122927 | 64 | 100 | 1-64/64 | toxin-antitoxin system HicB antitoxin [*K. pneumoniae*] |
| *primase* | 124739-126874 | 711 | 100 | 1-711/711 | DNA primase [*K. pneumoniae*] |
| *hp4* | 128377-129543 | 388 | 100 | 1-388/388 | phage major capsid protein [*K. pneumoniae*] |
| *hp5* | 131395-131730 | 111 | 100 | 1-111/111 | head-tail adaptor [*K. pneumoniae*] |
| *dusB* | 136380-135415 | 321 | 100 | 1-321/321 | tRNA dihydrouridine synthase DusB [*K. pneumoniae*] |
| *panF* | 139082-137631 | 483 | 100 | 1-483/483 | sodium/panthothenate symporter [*Klebsiella pneumoniae*] |
| *accC* | 140774-139425 | 449 | 100 | 1-449/449 | acetyl-CoA carboxylase biotin carboxylase [*K. pneumoniae*] |
| *accB* | 141252-140785 | 155 | 100 | 1-155/155 | acetyl-CoA carboxylase biotin carboxyl [*K. pneumoniae*] |
| *aroQ* | 141727-141275 | 150 | 100 | 1-150/150 | type II 3-dehydroquinate dehydratase [*K. pneumoniae*] |
| *msrQ* | 142559-141951 | 202 | 100 | 1-202/202 | protein-methionine-sulfoxide reductase MsrQ [*K. pneumoniae*] |
| *msrP* | 143560-142559 | 333 | 100 | 1-333/333 | protein-methionine-sulfoxide reductase MsrP [*K. pneumoniae*] |
| *csrD* | 144060-146000 | 646 | 100 | 1-646/646 | RNase E specificity factor CsrD [*K. pneumoniae*] |
| *mreC* | 147420-148412 | 330 | 100 | 1-330/330 | rod shape-determining protein MreC [*K. pneumoniae*] |
| *mreD* | 148412-148900 | 162 | 100 | 1-162/162 | rod shape-determining protein MreD [*K. pneumoniae*] |
| *maf* | 148908-149489 | 193 | 100 | 1-193/193 | septum formation inhibitor Maf [*K. pneumoniae*] |
| *rng* | 149492-150961 | 489 | 100 | 1-489/489 | ribonuclease E/G [*K. pneumoniae*] |
| *hp6* | 150999-154796 | 1265 | 100 | 1-1265/1265 | DUF3971 domain-containing protein [*K. pneumoniae*] |
| *hp7* | 154885-156330 | 481 | 100 | 1-481/481 | metalloprotease TldD [*K. pneumoniae*] |
| *reg* | 157295-156366 | 309 | 100 | 1-309/309 | transcriptional regulator [*K. pneumoniae*] |
| *aaeA* | 157638-158570 | 310 | 100 | 1-310/310 | p-hydroxybenzoic acid efflux pump AaeA [*K. pneumoniae*] |
| *aaeB* | 158576-160543 | 655 | 100 | 1-655/655 | p-hydroxybenzoic acid efflux pump AaeB [*K. pneumoniae*] |
| *deh* | 162838-163776 | 312 | 100 | 1-312/312 | malate dehydrogenase [*K. pneumoniae*] |
| *degS* | 164971-163913 | 352 | 100 | 1-352/352 | serine endoprotease DegS [*K. pneumoniae*] |
| *degQ* | 166426-165059 | 455 | 100 | 1-455/455 | serine endoprotease DegQ [*K. pneumoniae*] |
| *zapE* | 167189-168316 | 375 | 100 | 1-375/375 | cell division protein ZapE [*K. pneumoniae*] |
| *tnpA* | 175383-172417 | 988 | 100 | 1-988/988 | Tn*6346* transposase [*K. pneumoniae*] |
| *tnpA* | 176220-175504 | 238 | 100 | 1-238/238 | IS*26* transposase [*Klebsiella pneumoniae*] |
| *traX* | 176453-177199 | 248 | 100 | 1-248/248 | conjugal transfer protein TraX [*K. pneumoniae*] |
| *finO* | 177254-177814 | 186 | 100 | 1-186/186 | conjugal transfer fertility protein FinO [*K. pneumoniae*] |
| *hp8* | 178505-179131 | 208 | 100 | 1-208/208 | hypothetical protein [*K. pneumoniae*] |

^a^aa, amino acids.

^b^Query alignment region: match alignment region/total aa of the match.
