# Supplementary material for: Characterization of a Multidrug-Resistant Porcine Klebsiella pneumoniae Sequence Type 11 Strain Coharboring blaKPC-2 and fosA3 on Two Novel Hybrid Plasmids
Source: mSphere. 2019 Sep 11;4(5):e00590-19. doi: 10.1128/mSphere.00590-19 (PMC6739495; doi:10.1128/mSphere.00590-19)
Supplement: TABLE S2 [file mSphere.00590-19-st002.docx]

Table S2. Annotation of ORFs in plasmid pK15-FOS

| **Gene designation** | **location (start-end)** | **size (aa^a^)** | **aa identity (%)** | **Alignment region of the match** | **homologue description** |
| --- | --- | --- | --- | --- | --- |
| *repB* | 1-867 | 288 | 100 | 1-288/288 | RepB family plasmid replication protein [*K. peumoniae*] |
| *parA* | 1977-3182 | 401 | 100 | 1-401/401 | partitioning protein ParA [*K. peumoniae*] |
| *parB* | 3179-4156 | 325 | 100 | 1-325/325 | partitioning protein ParB [*K. peumoniae*] |
| *umuC* | 5509-4238 | 423 | 100 | 1-423/423 | DNA polymerase V subunit UmuC [*K. peumoniae*] |
| *umuD* | 5940-5509 | 143 | 100 | 1-143/143 | DNA polymerase V subunit UmuD [*K. peumoniae*] |
| *retA* | 6347-7834 | 495 | 100 | 1-495/495 | RetA [*K. peumoniae*] |
| *stbA* | 8083-9054 | 323 | 100 | 1-323/323 | putative prophage partitioning protein [*K. peumoniae*] |
| *hp1* | 9057-9728 | 223 | 100 | 1-223/223 | mediator of plasmid stability [*K. peumoniae*] |
| *met* | 10530-11159 | 209 | 100 | 1-209/209 | DNA methylase [*K. peumoniae*] |
| *hp2* | 11863-12630 | 255 | 100 | 1-255/255 | hypothetical protein [*K. peumoniae*] |
| *ardA* | 13782-14288 | 168 | 100 | 1-168/168 | antirestriction protein ArdA [*K. peumoniae*] |
| *hp3* | 16600-17955 | 451 | 100 | 1-451/451 | hypothetical protein [*K. peumoniae*] |
| *met* | 18003-18566 | 187 | 100 | 1-187/187 | SAM-dependent methyltransferase [*K. peumoniae*] |
| *ssb* | 19399-19941 | 180 | 100 | 1-180/180 | single-stranded DNA-binding protein [*K. peumoniae*] |
| *hp4* | 20308-22365 | 685 | 100 | 1-685/685 | hypothetical protein [*K. peumoniae*] |
| *psiB* | 22410-22841 | 143 | 100 | 1-143/143 | plasmid SOS inhibition protein B [*K. peumoniae*] |
| *psiA* | 22838-23566 | 242 | 100 | 1-242/242 | plasmid SOS inhibition protein A [*K. peumoniae*] |
| *toxin* | 28135-28455 | 106 | 100 | 1-106/106 | type II toxin-antitoxin system RelE/ParE toxin [*K. peumoniae*] |
| *reg* | 28445-28723 | 92 | 100 | 1-92/92 | XRE family transcriptional regulator [*K. peumoniae*] |
| *tnpA* | 30337-29633 | 234 | 100 | 1-234/234 | IS26 transposase [*K. peumoniae*] |
| Δ*traC* | 32638-30404 | 744 | 100 | 1-744/744 | truncated type-IV secretion system protein TraC [*K. peumoniae*] |
| *traR* | 33019-32798 | 73 | 100 | 1-73/73 | protein TraR [*K. peumoniae*] |
| *traV* | 33669-33154 | 171 | 100 | 1-171/171 | type IV conjugative transfer system TraV [*K. peumoniae*] |
| *trbG* | 33917-33666 | 83 | 100 | 1-83/83 | conjugal transfer protein TrbG [*K. peumoniae*] |
| *trbD* | 34159-33929 | 76 | 100 | 1-76/76 | conjugal transfer protein TrbD [*K. peumoniae*] |
| *traP* | 34703-34113 | 196 | 100 | 1-196/196 | conjugal transfer protein TraP [*K. peumoniae*] |
| *traB* | 36120-34693 | 475 | 100 | 1-475/475 | conjugal transfer protein TraB [*K. peumoniae*] |
| *traK* | 36791-36120 | 223 | 100 | 1-223/223 | type-F conjugative transfer secretin TraK [*K. peumoniae*] |
| *traE* | 37401-36835 | 188 | 100 | 1-188/188 | type IV conjugative transfer system TraE [*K. peumoniae*] |
| *traL* | 37734-37423 | 103 | 100 | 1-103/103 | type IV conjugative transfer protein TraL [*K. peumoniae*] |
| *traA* | 38114-37749 | 121 | 100 | 1-121/121 | type IV conjugative transfer system pilin TraA [*K. peumoniae*] |
| *traY* | 38375-38148 | 75 | 100 | 1-75/75 | relaxosome protein TraY [*K. peumoniae*] |
| *traJ* | 39215-38469 | 248 | 100 | 1-248/248 | conjugal transfer protein TraJ [*K. peumoniae*] |
| *hp5* | 40006-40653 | 215 | 100 | 1-215/215 | lytic transglycosylase [*K. peumoniae*] |
| *hok* | 43251-43093 | 52 | 100 | 1-52/52 | protein Hok [*K. peumoniae*] |
| *sok* | 43331-43510 | 59 | 100 | 1-59/59 | protein Sok [*K. peumoniae*] |
| *psiA* | 44250-43531 | 239 | 100 | 1-239/239 | plasmid SOS inhibition protein A [*K. peumoniae*] |
| *psiB* | 44681-44247 | 144 | 100 | 1-144/144 | plasmid SOS inhibition protein B [*K. peumoniae*] |
| *ssb* | 47691-47125 | 188 | 100 | 1-188/188 | single-stranded DNA-binding protein [*K. peumoniae*] |
| *met2* | 49058-48495 | 187 | 100 | 1-187/187 | class I SAM-dependent methyltransferase [*K. peumoniae*] |
| *hp6* | 50328-49105 | 407 | 100 | 1-407/407 | DUF3560 domain-containing protein [*K. peumoniae*] |
| *ltrA* | 52148-53815 | 555 | 100 | 1-555/555 | group II intron reverse transcriptase/maturase [*K. peumoniae*] |
| *hp7* | 55211-54786 | 141 | 100 | 1-141/141 | antirestriction protein [*K. peumoniae*] |
| *met2* | 55756-55184 | 190 | 100 | 1-190/190 | DNA methylase [*K. peumoniae*] |
| *parM* | 58326-59288 | 320 | 100 | 1-320/320 | plasmid segregation protein ParM [*K. peumoniae*] |
| *stbB* | 59291-59641 | 116 | 100 | 1-116/116 | protein StbB [*K. peumoniae*] |
| *tnpA* | 61987-62691 | 234 | 100 | 1-234/234 | IS26 transposase [*K. peumoniae*] |
| *bla*_TEM-1_ | 63303-64163 | 286 | 100 | 1-286/286 | TEM-1 [*E. coli*] |
| *rmtB* | 64333-65088 | 377 | 100 | 1-377/377 | Gentamicin-resistance methyltransferase RmtB [*E. coli*] |
| *tnpA* | 65214-65918 | 234 | 100 | 1-234/234 | IS26 transposase [*K. peumoniae*] |
| Δ*orf3* | 65971-66399 | 142 | 100 | 1-142/142 | transcriptional regulator, *tetR* family [*K. peumoniae*] |
| *orf2* | 66418-67206 | 262 | 100 | 1-262/262 | winged helix family transcriptional regulator [*K. peumoniae*] |
| *orf1* | 67206-67724 | 172 | 100 | 1-172/172 | hypothetical protein [*K. peumoniae*] |
| *fosA3* | 68145-67729 | 138 | 100 | 1-138/138 | Fosfomycin resistance protein FosA3 [*K. peumoniae*] |
| *tnpA* | 68531-69235 | 234 | 100 | 1-234/234 | IS26 transposase [*K. peumoniae*] |
| Δ*bla*_TEM-1_ | 69291-70028 | 245 | 100 | 1-245/245 | truncated TEM-1 [*E. coli*] |
| *orf477* | 70287-70763 | 158 | 100 | 1-158/158 | Tryptophan synthase beta chain like-protein [*E. coli*] |
| *bla*_CTX-M-55_ | 71685-70810 | 291 | 100 | 1-291/291 | CTX-M-55 [*E. coli*] |
| *tnpA* | 72811-72107 | 234 | 100 | 1-234/234 | IS26 transposase [*K. peumoniae*] |
| *tnpR* | 73063-72875 | 62 | 100 | 1-62/62 | resolvase of Tn2 [*E. coli*] |
| *tnpA* | 74072-73368 | 234 | 100 | 1-234/234 | IS26 transposase [*K. peumoniae*] |
| *gshB* | 74312-75775 | 487 | 100 | 1-487/487 | Glutathione synthase [*E. coli*] |
| *tnpA* | 76193-77197 | 334 | 100 | 1-334/334 | IS5075 transposase [*E. coli*] |
| Δ*repA* | 78417-77770 | 215 | 100 | 1-215/215 | truncated replication initiation protein IncN RepA [*E. coli*] |
| *tnpA* | 79127-78423 | 234 | 100 | 1-234/234 | IS26 transposase [*K. peumoniae*] |
| *exc1* | 79438-79887 | 149 | 100 | 1-149/149 | Putative entry exclusion protein 1 [*E. coli*] |
| *lip* | 80535-81074 | 179 | 100 | 1-179/179 | putative lipoprotein [*E. coli*] |
| *met* | 83034-85082 | 682 | 100 | 1-682/682 | Type III restriction-modification methylation subunit [*E. coli*] |
| *res* | 85087-87723 | 878 | 100 | 1-878/878 | Type III restriction-modification endonuclease Res [*E. coli*] |
| *mobX* | 88350-87766 | 194 | 100 | 1-194/194 | relaxation protein [*E. coli*] |
| *mobA* | 89100-88363 | 245 | 100 | 1-245/245 | mobilization protein [*E. coli*] |
| *repA* | 92207-91521 | 228 | 100 | 1-228/228 | truncated replication initiation protein IncN RepA [*E. coli*] |
| *tnpA* | 92959-92255 | 234 | 100 | 1-234/234 | IS26 transposase [*K. peumoniae*] |
| *reg* | 93481-93158 | 107 | 100 | 1-107/107 | ArsR family transcriptional regulator [*E. coli*] |
| *ydaA* | 93586-94623 | 345 | 100 | 1-345/345 | YdaA [*E. coli*] |
| *finO* | 95533-94916 | 205 | 100 | 1-205/205 | IncI1 conjugal transfer repressor protein FinO [*E. coli*] |
| *SMC* | 97281-95725 | 518 | 100 | 1-518/518 | chromosome segregation protein SMC [*E. coli*] |
| *hp8* | 98134-97544 | 196 | 100 | 1-196/196 | hypothetical protein [*E. coli*] |
| *ydfA* | 98391-98134 | 85 | 100 | 1-85/85 | YdfA protein [*E. coli*] |
| *hp9* | 98745-100883 | 712 | 100 | 1-712/712 | hypothetical protein [*E. coli*] |
| *pin* | 101461-101045 | 138 | 100 | 1-138/138 | PIN domain-containing protein [*E. coli*] |
| *vagD* | 101688-101458 | 76 | 100 | 1-76/76 | virulence-associated protein VagD [*E. coli*] |
| *vagC* | 101984-102274 | 96 | 100 | 1-96/96 | virulence-associated protein VagC [*E. coli*] |
| *exc* | 105438-103213 | 741 | 100 | 1-741/741 | phage T7 exclusion protein [Salmonella enterica] |
| *reg* | 106528-105440 | 362 | 100 | 1-362/362 | putative transcriptional regulator [*E. coli*] |
| *hp10* | 109328-110332 | 334 | 100 | 1-334/334 | hypothetical protein [*K. peumoniae*] |
| *res* | 110570-111295 | 241 | 100 | 1-241/241 | Resolvase [*K. peumoniae*] |
| ^a^aa, amino acids.  ^b^Query alignment region: match alignment region/total aa of the match. | | | | | |
